# Adiponectin inhibits lipoplysaccharide-induced inflammation and promotes osteogenesis in hPDLCs

**DOI:** 10.1042/BSR20192668

**Published:** 2021-03-02

**Authors:** Huan-huan Wu, Yuan Guo, Yin-fei Pu, Zhi-hui Tang

**Affiliations:** 1 Second Dental Center, School and Hospital of Stomatology, Peking University, Beijing 100081, People’s Republic of China; 2 National Engineering Laboratory for Digital and Material Technology of Stomatology, School and Hospital of Stomatology, Peking University, Beijing 100081, People’s Republic of China

**Keywords:** adiponectin, human periodontal ligament cells, inflammation, lipopolysaccharide, osteogenic differentiation

## Abstract

Periodontal diseases are infections of the structures that surround and support the teeth; they are characterized by local inflammation and alveolar bone loss. Most treatments focus on only one aspect, inhibiting inflammation, or promoting osteoblasts. We set out to develop a new method that would intervene in the two aspects simultaneously. Adiponectin (APN), secreted by adipocytes, inhibits the inflammatory response and promotes osteogenesis. However, its role in human periodontal ligament cells (hPDLCs) is unclear. Therefore, we aim to investigate whether APN could suppress lipopolysaccharide (LPS)-induced inflammation and promote osteogenesis in hPDLCs. In the present study, we stimulated hPDLCs with LPS in the presence or absence of APN. Real-time PCR and Western blotting results demonstrated that APN partially inhibited the activation of the classical nuclear factor κ-B (NF-κB) pathway. These results were confirmed by a change of expressions of NF-κB downstream inflammatory genes, such as decreased cyclooxygenase (COX)-2 and tumor necrosis factor α (TNF-α), along with increased interleukin (IL)-10. As for the role of APN in osteogenesis, Alizarin Red S staining showed that APN treatment induced more calcium deposition nodules than controls. We also found that APN enhanced the expression of osteoblast-related genes (osteopontin (OPN), collagen 1, osteocalcin, alkaline phosphatase, runt-related transcription factor 2 (RUNX2), and bone morphogenetic protein 2) in hPDLCs via the APPL1 (the adaptor protein containing PH domain, PTB domain, and leucine zipper motif 1)/p38 signal transduction pathway. Therefore, APN inhibits LPS-induced inflammation and promotes osteogenesis in hPDLCs and may have potential therapeutic value in treating periodontitis by inhibiting the inflammatory lesions and contributing to bone tissue regeneration.

## Introduction

Periodontal diseases such as gingivitis and periodontitis are caused by pathogenic bacteria [1]. Periodontal infections are associated with systemic diseases (such as type 2 diabetes) and obesity [2,3]. Lipopolysaccharide (LPS) produced by oral microbiota is considered to be the major cause of periodontitis [4,5]. LPS and pathogenic bacteria induce a host inflammatory response, which involves many inflammatory mediators, such as interleukin (IL)-1, IL-10, tumor necrosis factor α (TNF-α), and cyclooxygenase (COX)-2 in the periodontal tissues [6]. The inflammatory response results in periodontal ligament damage, alveolar bone resorption, and even tooth loss. The major challenge in contemporary periodontal therapy is to promote periodontal tissue regeneration. The ligament is the most important component of periodontal tissue, being the connective tissue between two hard tissues, cementum of teeth and alveolar bone. Human periodontal ligament cells (hPDLCs) are a heterogeneous group of cells containing mesenchymal stem cells [7,8]. The potential for multidirectional differentiation of hPDLCs is essential for periodontal tissue regeneration. Many studies have investigated the osteogenic differentiation of hPDLCs [9,10]. In order to promote periodontal tissue regeneration, several methods such as mixed cells, scaffolds, and growth factors have been applied [11].

Adiponectin (APN) secreted by adipocytes, has both immune and metabolic functions; it has anti-inflammatory, insulin-sensitizing, anti-atherosclerotic, and anti-diabetic properties [12,13]. Many experiments have shown that serum APN is anti-inflammatory in atherosclerosis and rheumatoid arthritis by inhibiting the nuclear factor κ-B (NF-κB) pathway [14]. Besides its anti-inflammatory effect, most *in vitro* studies have shown that APN plays an important role in the regulation of bone metabolism through promoting the osteoblastic differentiation and suppressing osteoclast formation [15,16].

APN performs function by binding its receptors, adipoR1 and adipoR2, which have been found in periodontal tissues [17]. APPL1 (adaptor protein containing PH domain, PTB domain and leucine zipper motif-1), downstream adaptor protein containing multiple protein–protein interaction domains, interacts with AdiopR1 [17]. Then they activate the p38 mitogen-activated protein kinase (MAPK) signaling pathways [18,19]. The p38 MAPK is a major kinase in the MAPK family and acts as an essential mediator in regulating APN-induced osteogenic differentiation of Human Jaw Bone Marrow Mesenchymal Stem Cell [16,20]. Additionally, APN is also involved in periodontitis-related systemic conditions such as type 2 diabetes mellitus (T2DM) and obesity [21,22]. Diabetes mellitus and obesity contribute to the severity of periodontitis and the compromised healing after periodontal therapy [23,24]. Recent studies have reported that decreased levels of APN, as found in type 2 diabetes and obesity, may compromise periodontal health and healing. APN is produced by hPDLCs and could accelerate wound closure [25]. In this light, it is necessary to investigate the anti-inflammation and osteogenic differentiation effect of APN on hPDLCs.

## Materials and methods

### hPDLCs culture and osteogenic induction

hPDLCs were obtained by two ways including a purchase from company (hPDLCs1) and primary cultured by our lab (hPDLCs2). All the experiments were repeated using these cell lines. The commercial cell line (hPDLCs1) was sourced from ScienCell Co. (San Diego, CA, U.S.A., Cat No. 2630). The primary culture (hPDLCs2) was according to the method of Somerman et al. [26]. Ethical approval had been obtained from the Ethics Committee of Peking University Hospital of Stomatology with the following reference number PKUSSIRB-201310067. The study was performed in accordance with the Declaration of Helsinki. Intact caries-free, freshly extracted premolars (*n*=5) were collected from five patients (12–13 years old) in the Department of Orthodontics, Peking University Hospital of Stomatology. Informed consent was obtained from all the patients and their parents. hPDLCs were isolated from the mid-third of the teeth roots avoiding contamination from the gingival and dental pulp tissues. hPDLCs were cultured in minimum essential medium-α (α-MEM) supplemented with 10% fetal bovine serum (FBS) at 37°C in a CO_2_ incubator. hPDLCs are characterized by their spindle morphology and immunofluorescent method with antibody to fibronectin. hPDLCs were negative for HIV-1, HBV, HCV, mycoplasma, bacteria, yeast and fungi. For the subsequent experiments, cells from the third passage were used. Osteoblast differentiation was induced using conditional medium containing α-MEM, 100 U/ml penicillin, 10% FBS, 100 mg/ml streptomycin, 10 nM dexamethasone, 10 mM β-glycerophosphate, and 50 μg/ml l-ascorbic acid (Sigma–Aldrich, St. Louis, MO, U.S.A.). The cells were seeded in 60-mm dishes and cultured with or without 1 μg/ml APN (Z03072; Genscript, Piscataway, NJ, U.S.A.) for 7 days to examine ossification-related gene expression or for 21 days to assess nodule formation by Alizarin Red S staining. For the mechanistic analyses, cells were starved for 12 h in α-MEM without FBS and then treated with or without 1 μg/ml APN [27].

LPS from *Porphyromonas gingivalis* (LPS-PG; Invivogen, San Diego, CA, U.S.A.) was used to induce the inflammation of hPDLCs (hPDLCs1 and hPDLCs2). hPDLCs (hPDLCs1 and hPDLCs2) were plated at a density of 5000 cell/cm^2^ for 24 h at 37°C at 5% CO_2_ and then cells were exposed to 5 or 10 µg/ml LPS for 12 h. Real-time PCR was performed to investigate the changes of pro- and anti-inflammatory cytokine levels. To investigate the anti-inflammation of APN, the cells were pretreated with/without APN (1 µg/ml) for 1 h prior to LPS (10 µg/ml) exposure for 12 h.

Osteoblast differentiation was induced using osteogenic medium containing α-MEM, 100 U/ml penicillin, 10% FBS, 100 mg/ml streptomycin, 10 nM dexamethasone, 10 mM β-glycerophosphate, and 50 μg/ml l-ascorbic acid (Sigma–Aldrich, St. Louis, MO, U.S.A.). The cells (5000 cell/cm^2^) were seeded in 60-mm dishes for 24 h and cultured with or without 1 μg/ml APN (Z03072; Genscript, Piscataway, NJ, U.S.A.) for 7 days to examine osteogenic-related gene expression or for 21 days to assess nodule formation by Alizarin Red S staining. For the mechanistic analyses, cells were starved for 12 h in α-MEM without FBS and then treated with or without 1 μg/ml APN for 1 week.

### RNA extraction, reverse-transcription PCR, and real-time PCR

Total RNA was isolated from hPDLCs (hPDLCs1 and hPDLCs2) using the TRIzol reagent (Invitrogen, Carlsbad, CA, U.S.A.). Complementary DNA (cDNA) was synthesized and amplified using a PrimeScript First Strand cDNA Synthesis kit (TaKaRa, Minato-ku, Tokyo, Japan) according to the manufacturer’s instructions. To examine inflammatory cytokines and osteogenesis-related genes expression, real-time PCR was performed using the Power SYBR Green PCR Master Mix and an ABI PRISM 7500 sequence detection system (Applied Biosystems, Foster City, CA, U.S.A.). All primer sequences were listed in Table 1. The reaction consists of 25 ng template cDNA, 1× SYBR Green PCR, 150 nM each of forward and reverse primer in a 20 μl final volume per tube. Reaction components are pre-mixed in eight-well PCR tube-strips. The thermal condition of the PCR was 50°C for 2 min, 95°C for 10 min, followed by 40 cycles at 95°C for 15 s, and 60°C for 1 min. The comparative CT (2^−ΔΔ*C*_T_^) method was employed to evaluate fold gene expression differences between groups. The gene expression levels were normalized to that of the housekeeping gene *GAPDH*.

**Table 1:** Primers used for real time RT-PCR

Gene	Forward primer	Reverse primer
*GAPDH*	CAATGACCCCTTCATTGACC	TGGACTCCACGACGTACTCA
*RUNX-2*	ACTACCAGCCACCGAGACCA	ACTGCTTGCAGCCTTAAATGACTCT
*OCN*	AGCCACCGAGACACCATGAGA	AGCCACCGAGACACCATGAGA
*ALP*	AACATCAGGGACATTGACGTG	GTATCTCGGTTTGAAGCTCTTCC
*OPN*	CATACAAGGCCATCCCCGTT	ACGGCTGTCCCAATCAGAAG
*COL1A1*	AGACACTGGTGCTAAGGGAGA	GACCAGCAACACCATCTGCG
*IL-10*	ACTTTAAGGGTTACCTGGGTTGC	TCACATGCGCCTTGATGTCTG
*COX-2*	CTGGCGCTCAGCCATACAG	ACACTCATACCATACACCTCAAT
*TNF-α*	CAGAGGGAAGAGTTCCCCAG	CCTCAGCTTGAGGGTTTGCTAC

### Alizarin Red S staining and mineralization assays

hPDLCs (hPDLCs1 and hPDLCs2) were seeded in 60-mm dishes at the indicated densities and cultured for 21 days in osteoinductive medium. The conditional medium was replaced every 2 days, and APN was added accordingly at a concentration of 1 µg/mL. Alizarin Red S staining was at room temperature on day 21 of osteoinduction [28,29]. The results were analyzed using ImageJ software (National Institutes of Health, Bethesda, MD, U.S.A.). To quantify matrix mineralization, samples were incubated in 100 mM cetylpyridinium chloride for 1 h, after which the absorbance of the released Alizarin Red S staining was measured at 562 nm by using a microplate reader (model 680, Bio-Rad).

### Western blot analysis

hPDLCs (hPDLCs1 and hPDLCs2) were cultured in 60-mm dishes for 1 week, starved for 12 h after reaching 90% confluence, and then treated with APN three times (once every 2 days) during the culture period. Total protein was extracted using RIPA buffer (CW2333S; Applygen, Beijing, China) and the concentration was measured with bicinchoninic acid reagent (Thermo Fisher Scientific, MA, U.S.A.). Protein samples (40 mg/lane) in loading buffer were separated by SDS/PAGE and transferred on to a polyvinylidene difluoride membrane (Millipore, MA, U.S.A.). The primary antibodies used were as follows: anti-GAPDH (g0314; Santa Cruz Biotechnology, California, U.S.A.), anti-runt-related transcription factor 2 (RUNX2) (8486S; Cell Signaling Technology (CST), MA, U.S.A.), anti-P-p38 (4511; CST), anti-p38 (8690; CST), anti-P-p65 (3033; CST), anti-p65 (3034; CST), anti-APPL1 (3858S; CST) at 1:1000. Anti-rabbit secondary antibody (7074S; CST) at 1:5000 was used as described previously [16]. The Western blots were scanned using an Odyssey® CLx Infrared Imaging System to visualize the protein bands and were analyzed using ImageJ software (National Institutes of Health).

### Small interfering RNA-mediated knockdown of APPL1 in hPDLCs

Small interfering RNA (siRNA) sequences were used to knock down APPL1 in hPDLCs (hPDLCs1) using Lipofectamine®2000 Transfection Reagent according to the manufacturer’s instructions (Thermo Scientific, Rockford, US). hPDLCs1 were cultured in 60-mm dishes and transfected with 100 nM scrambled or APPL1 siRNA for 12 h. Cultures with transduction efficiencies of >90% were used in the analyses. The siRNA sequence was CAGTCAGAAGAGAGTGATT, and the control sequence was TTCTCCGAACGTGTCACGT (GeneChem, Shanghai, China).

### Blockade of p38 MAPK phosphorylation by SB202190

The cells for the mechanistic analyses were starved for 12 h in α-MEM without FBS and then treated with or without 1 μg/ml APN [27]. Cells were treated with the p38 MAPK inhibitor SB202190 (DMSO vehicle; Selleck, Houston, TX, U.S.A.) for 2 h before APN treatment as noted. The cells (5000 cell/cm^2^) were seeded in 60-mm dishes for 7 days to examine osteogenic-related gene expression or for 21 days to assess nodule formation by Alizarin Red S staining.

### Statistical analysis

All experiments were carried out in triplicate. Data expressed as mean ± standard deviation were obtained from three independent researches. The results were analyzed for statistical significance using Student’s *t* test. Statistically significant differences (*P*<0.05) among the various groups were evaluated using one-way ANOVA. Differences were considered significant at *P*<0.05. All statistical analyses were performed using SPSS 19.0 software (SPSS Inc., Chicago, IL, U.S.A.).

## Results

### LPS triggered the inflammatory response in hPDLCs

**Figure 1:**
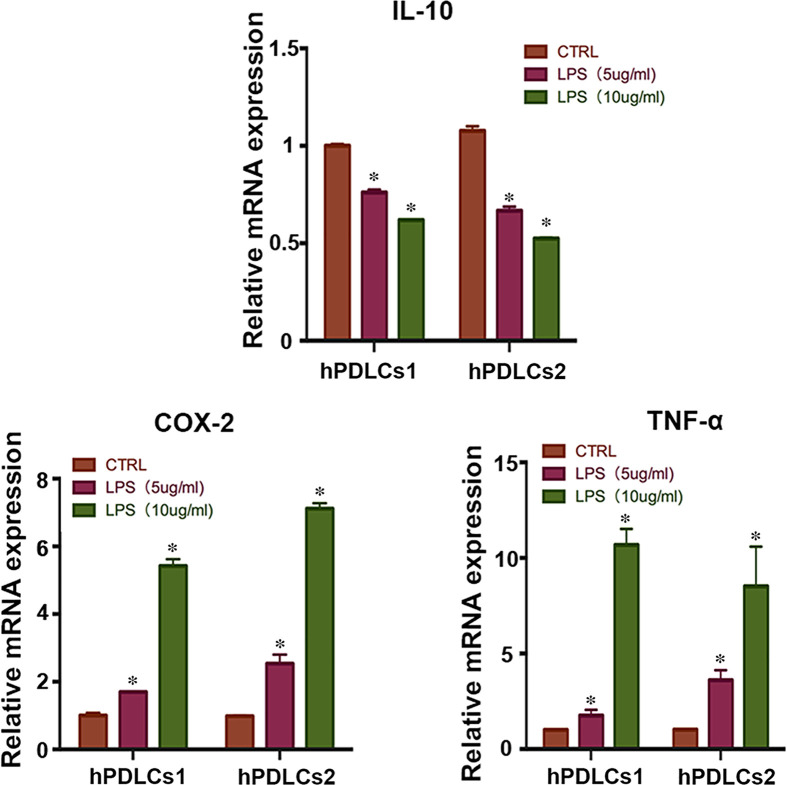
Gene expression profile of inflammatory cytokines in hPDLCs cultured with LPS Histograms of the relative mRNA expression of pro-inflammatory (COX-2 and TNF-α) and anti-inflammatory (IL-10) cytokines after exposure of hPDLCs (hPDLCs1 and hPDLCs2) to LPS (5 or 10 µg/ml) for 12 h (mean ± SD, *P<0.05, experiments performed in triplicate and repeated three times independently).

To determine the appropriate concentration of LPS needed to induce the inflammatory response, hPDLCs (hPDLCs1 and hPDLCs2) were exposed to different concentrations for 12 h and real-time PCR was performed to investigate the changes of pro- and anti-inflammatory cytokine levels. The results showed that 5 and 10 µg/ml LPS improved the expression of pro-inflammatory cytokines COX-2 (increased to 1.7-fold, 5.4-fold) and TNF-α (increased to 1.8-fold, 10.7-fold), whereas suppressed the expression of anti-inflammatory cytokine IL-10 (decreased to 75, 50%) compared with the control group (*P*<0.05) (Figure 1). In addition, the changes were dose-dependent (Figure 1). Therefore, 10 µg/ml LPS was chosen to trigger the inflammatory response of hPDLCs in the remaining experiments.

### APN reduced the expression of pro-inflammatory cytokines and promoted the expression of an anti-inflammatory cytokine in LPS-stimulated hPDLCs

**Figure 2:**
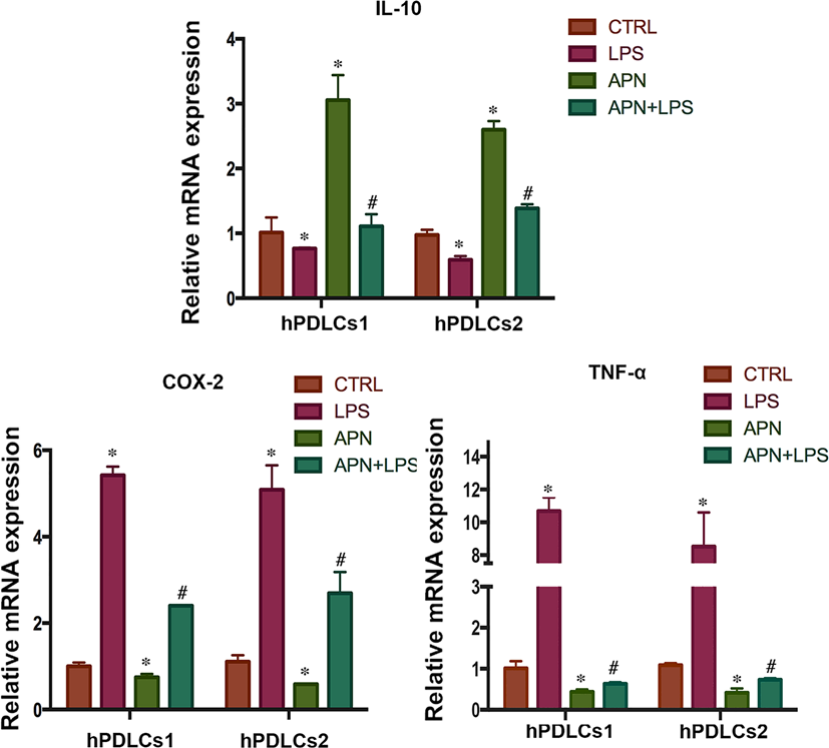
Effects of APN on the expression of IL-10, COX-2, and TNF-α in LPS-stimulated hPDLCs Relative of COX-2, TNF-α, and IL-10 mRNA expression in hPDLCs (hPDLCs1 and hPDLCs2) pretreated with APN (1 µg/ml) for 1 h before LPS (10 µg/ml) exposure for 12 h (*P<0.05 compared with control; ^#^*P*<0.05 compared with LPS group; experiments performed in triplicate and repeated three times independently).

To explore the effects of APN on the expression of inflammatory cytokines, hPDLCs (hPDLCs1 and hPDLCs2) were pretreated with APN (1 µg/ml) for 1 h before LPS (10 µg/ml) exposure for 12 h. Real-time PCR showed that LPS enhanced the expression of COX-2 and TNF-α and depressed that of IL-10, while APN suppressed COX-2 and TNF-α expression and induced IL-10 expression compared with control. In addition, pretreatment with APN suppressed the higher expression of COX-2 and TNF-α induced by LPS and enhanced the expression of IL-10 inhibited by LPS, compared with cells exposed to LPS alone (*P*<0.05) (Figure 2).

### APN suppressed the LPS-induced inflammatory response in hPDLCs via the NF-κB signaling pathway

**Figure 3:**
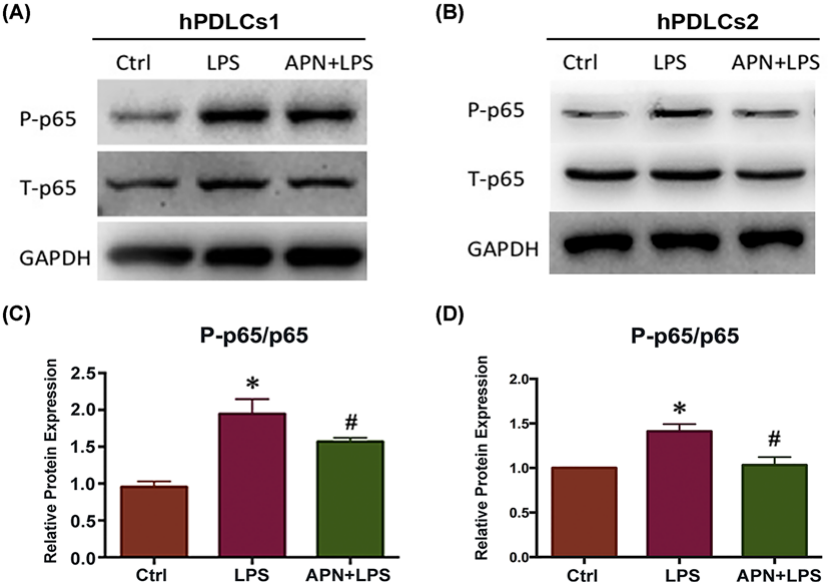
Effects of APN on phosphorylated NF-κB p65 (P-p65) in LPS-stimulated hPDLCs (**A,B**) Representative Western blots showing the nuclear expression of P-p65, total p65 (T-p65), and loading control (GAPDH) from control hPDLCs, and those exposed to LPS alone, and with APN (1 µg/ml) pretreatment for 1 h before LPS (10 µg/ml) exposure. (**C,D**) Quantitative analysis of relative protein expression as in (A,B) (mean ± SD; *P<0.05 compared with control; ^#^*P*<0.05 compared with LPS alone; experiments performed in triplicate and repeated three times independently).

We next investigated the molecular mechanism underlying the anti-inflammatory effect of APN. NF-κB is a key factor in regulating the inflammatory response. In the absence of an inflammatory stimulus, the NF-κB p65 subunit is localized in the cytoplasm. LPS activates NF-κB p65 and promotes its migration into the nucleus, initiating the expression of inflammatory cytokines [30]. We therefore investigated whether NF-κB p65 was involved in the APN anti-inflammatory process. hPDLCs (hPDLCs1 and hPDLCs2) were treated with or without APN (1 µg/ml) for 1 h prior to LPS (10 µg/ml) exposure for 1 h. Western blot analysis showed that LPS exposure led to rapid phosphorylation of the NF-κB p65 subunit (2.05-fold increase). It indicated that the NF-κB p65 subunit was phosphorylated and then transported from the cytoplasm to the nucleus after LPS treatment (Figure 3). APN pretreatment reduced the phosphorylation of NF-κB p65 induced by LPS (Figure 3).

### Osteogenesis-related gene expression in APN-treated hPDLCs

**Figure 4:**
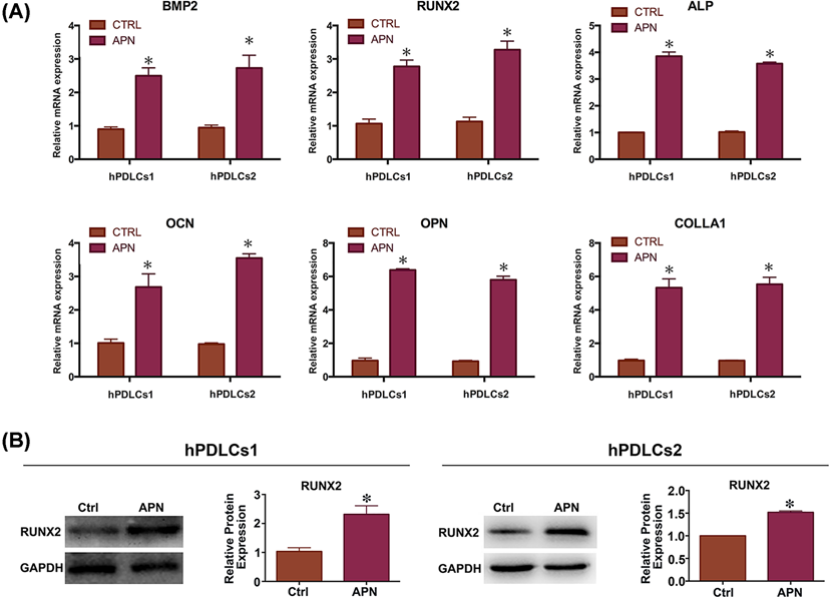
Effects of APN on the expression of ossification-related genes in hPDLCs hPDLCs were treated with APN (1 µg/ml) for 7 days under osteoinduction condition. (**A**) Relative expression of OPN, COL1A1, OCN, ALP, RUNX2, and BMP2 mRNAs in response to APN (*P<0.05 relative to control). (**B**). Representative Western blots RUNX2 in the 1 µg/ml APN group and quantitative analysis of the Western blots results (mean ± SD; *P<0.05 compared with control; experiments performed in triplicate and repeated three times independently).

To determine the osteogenic effect of APN on hPDLCs (hPDLCs1 and hPDLCs2), we evaluated ossification-related gene expression using real-time PCR after culturing the cells in osteoblast-inducing differentiation medium for 1 week. Real-time PCR showed that APN up-regulated the expression of BMP2, RUNX2, ALP, osteocalcin (OCN), osteopontin (OPN), collagen 1 (COL1A1) compared with the control group (*P*<0.05) (Figure 4A). Western blot analysis further confirmed that the expression of the osteogenesis-related genes RUNX2 in the 1 µg/ml APN group was higher (almost 2.5-fold, by densitometric scanning) than that in the control group (Figure 4B), further demonstrating the promoting osteo-differentiation ability of APN.

### Matrix mineralization of APN-treated hPDLCs

**Figure 5:**
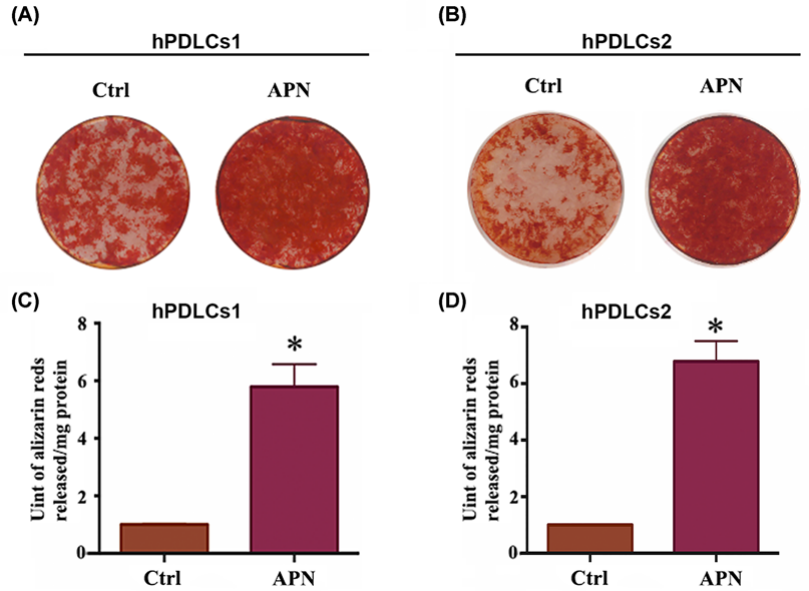
Alizarin Red S staining and mineralization assays on day 21 (**A,B**) Representative images of Alizarin Red S staining of control and Alizarin Red S-stained hPDLCs (hPDLCs1 and hPDLCs2) treated with 1 µg/ml APN on day 21. (**C,D**) Statistics for mineralization of hPDLCs (hPDLCs1 and hPDLCs2) treated with 1 µg/ml APN for 21 days (mean ± SD; *P<0.05 compared with control; experiments performed in triplicate and repeated three times independently).

After 21 days of osteoinduction, hPDLCs (hPDLCs1 and hPDLCs2) were stained with Alizarin Red S to assess the role of APN in the osteogenic differentiation of hPDLCs (hPDLCs1 and hPDLCs2). The results showed that matrix mineralization in the 1 µg/ml APN group was superior to that of the control group (Figure 5A). Furthermore, quantitation showed that 1 µg/ml APN-treated hPDLCs showed greater Alizarin Red S staining (5.79-fold) than the control group (Figure 5B).

### Activation of p38 MAPK phosphorylation by APN depended on APPL1 in hPDLCs

**Figure 6:**
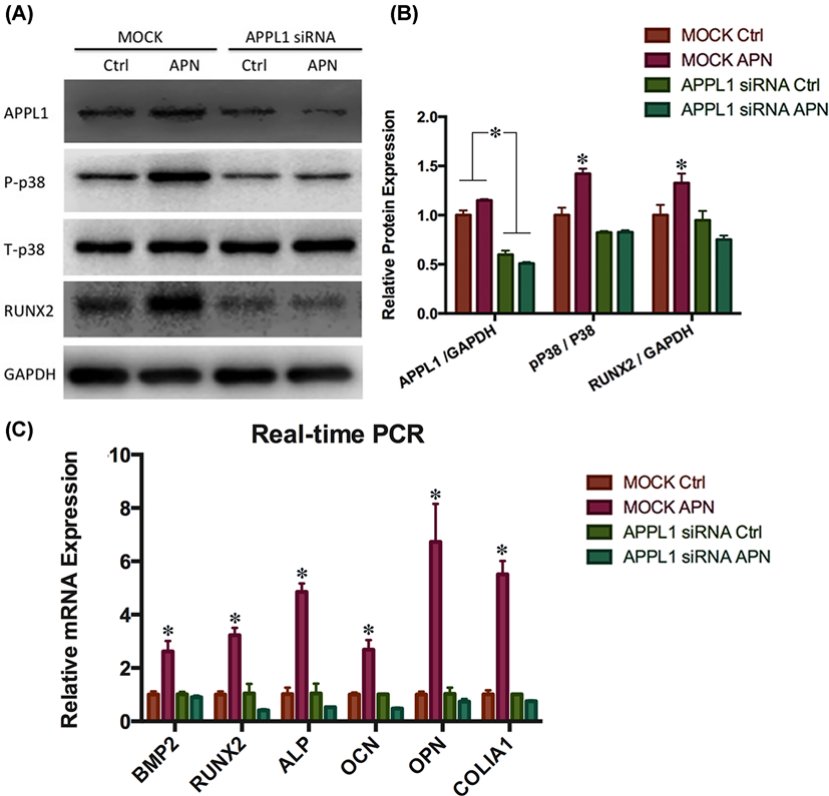
Activation of p38 MAPK phosphorylation by APN depended on APPL1 in hPDLCs (**A**) Representative Western blots of P-p38, total p38 (T-p38), APPL1, and RUNX2 in hPDLCs1 transfected with mock or APPL1 siRNA and treated with APN (1 µg/ml) for 7 days. (**B**) Quantitative analysis of data as in (A) (mean ± SD; *P<0.05 compared with control; experiments performed in triplicate). (**C**) Relative expression of OPN, COL1A1, OCN, ALP, RUNX2, and BMP2 mRNAs in response to APN in hPDLCs1transfected with mock or APPL1 siRNA and treated with APN (1 µg/ml) for 7 days (mean ± SD; *P<0.05 compared with control; experiments performed in triplicate and repeated three times independently).

Given that APN promoted osteogenic differentiation and mineralization in hPDLCs, we then investigated the underlying molecular mechanism. We used siRNA to knock down the expression of APPL1, a key regulator of the APN signaling pathway. Western blot results showed APPL1 expression decreased significantly after knockdown (Figure 6A). APN significantly enhanced the expression of phosphorylated p38 (P-p38) and RUNX2 in the mock group, while this effect was blocked in the APPL1-knockdown group (Figure 6A). There was no change in total p38 expression. Real-time PCR showed similar results that osteogenic-related genes (BMP2, RUNX2, ALP, OCN, OPN, and COL1A1) markedly increased in the APN-treated group, but did not change after APPL1 knockdown (Figure 6C). These results indicated that APN enhances the osteogenic differentiation of hPDLCs via the APPL1 pathway.

### Blocking phosphorylation of p38 MAPK inhibits osteogenesis of hPDLCs

**Figure 7:**
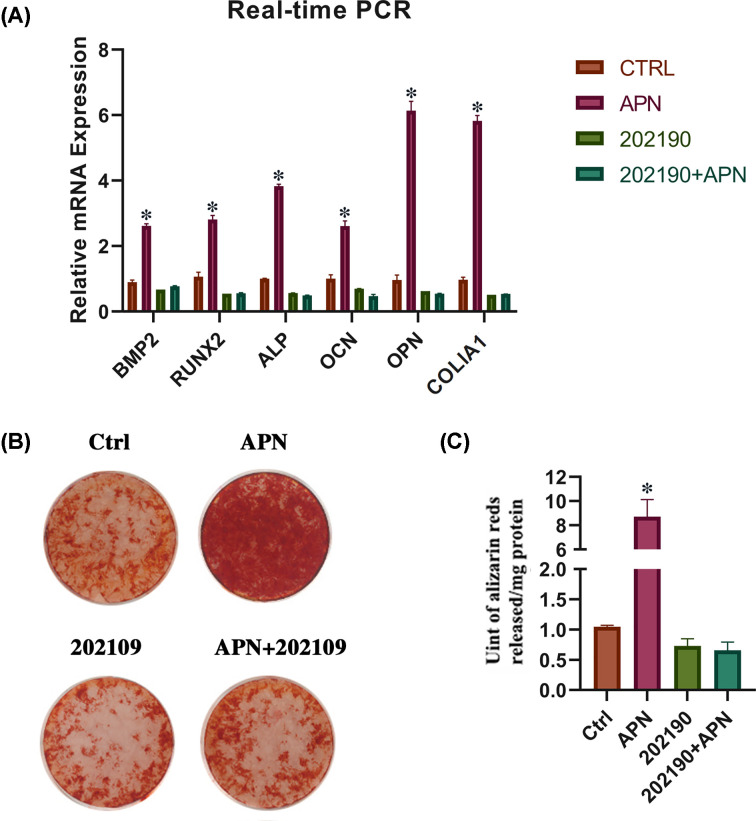
Inhibition of p38 MAPK phosphorylation weaken the osteogenesis induced by APN in hPDLCs (**A**) Relative expression of OPN, COL1A1, OCN, ALP, RUNX2, and BMP2 mRNAs in hPDLCs in different groups (control, APN, 202190, 202190+APN) on day 7 (mean ± SD; *P<0.05 compared with control; experiments performed in triplicate and repeated three times independently). (**B,C**) Representative images of Alizarin Red S staining of hPDLCs in different group (control, APN, 202190, 202190+APN) on day 21 (mean ± SD; *P<0.05 compared with control; experiments performed in triplicate and repeated three times independently).

We then explore the effect of p38 MAPK in the osteogenesis of hPDLCs. The p38 MAPK inhibitor 202190 was used to block phosphorylation of p38 MAPK. Real-time PCR showed that osteogenic-related genes (*BMP2, RUNX2, ALP, OCN, OPN*, and *COL1A1*) markedly increased in the APN-treated group. But in the cells pretreated with 202190, the expression of osteogenesis-related genes did not significantly increase (Figure 7A). Alizarin Red S staining showed similar results. And there were fewer positive red calcifying nodules were observed in the p38 MAPK inhibition group than in the other groups (Figure 7B,C).

## Discussion

In the present study, we have shown two important findings regarding the potential role of APN in treating periodontal disease. First, APN inhibited the NF-κB activation stimulated by LPS in hPDLCs and selectively regulated the expression of inflammatory genes, such as enhancing IL-10 and decreasing COX-2 and TNF expression. Second, APN promoted the expression of genes associated with bone formation (BMP2, RUNX2, ALP, OCN, OPN and COL1A1), and this was blocked by APPL1 siRNA. Furthermore, previous studies have shown that the serum APN levels are lower in patients with severe periodontitis, and markedly increase after periodontal intervention [31–39]. These results indicate that APN is involved in periodontal disease and that decreased APN expression in patients with periodontitis may be one of the causes of aggravated inflammation and decreased bone formation.

Periodontitis is initiated by microorganisms in dental plaque [40]. Among them, *P. gingivalis* is the most important pathogenic organism, and its LPS is thought to be a potent stimulus of inflammation [41,42]. LPS activates the NF-κB pathway and then initiates the subsequent inflammatory response, finally leading to periodontal tissue destruction [43]. Therefore, we selected LPS to stimulate HPDLCs, and found that it markedly increased the expression of pro-inflammatory cytokines (COX-2 and TNF-α) and significantly decreased the anti-inflammatory cytokine IL-10 in a concentration-dependent manner. However, inflammation is a complex process and LPS stimulation does not completely simulate the real inflammatory state; it only reflects a simplified condition of inflammation. Further clinical research is needed.

APN is known to play an important role in the inhibition of inflammation in many diseases such as atherosclerosis, cardiovascular disease, type 2 diabetes, and metabolic syndrome [31,32,44]. In this study, we first found that APN regulated the inflammatory response in HPDLCs; it inhibited the LPS-stimulated NF-κB activation and transport to the nucleus. This inhibition of NF-κB signaling then mediated the expression of downstream inflammatory cytokines. APN suppressed the expression of pro-inflammatory cytokines (COX-2 and TNF-α) induced by LPS. COX-2 is an inducible enzyme, which is not present in the healthy tissue but is expressed by cells involved in inflammatory processes. COX-2 is involved in the conversion of arachidonic acid into prostaglandins, which activate osteoclasts and promote bone absorption. TNF-α induces apoptosis in gingival epithelial cells in periodontitis. Therefore, decrease in the expression of these pro-inflammatory factors may be a mechanism by which APN reduces inflammatory injury. Moreover, APN promoted the expression of IL-10 in LPS-stimulated hPDLCs. IL-10 is an anti-inflammatory cytokine and controls the inflammatory response [45]. This is in line with the use of APN treatment in atherosclerosis; pretreatment with APN reduces LPS-stimulated TNF-α production and stimulates production of IL-10 in human and pig macrophages [46–50]. This indirect and direct evidence indicate that APN suppresses inflammation in periodontitis. However, the underlying mechanism has not been explained.

Apart from its anti-inflammatory effect, APN has been implicated in tissue regeneration, especially osteogenesis [16,51,52]. Many studies have improved the osteoblastic differentiation of hPDLCs [53–55]. However, the effects of APN on osteogenic differentiation of hPDLCs have received little attention. So, we stimulated hPDLCs with APN in osteogenic induction medium for 7 days and determined the expression of osteogenesis genes at mRNA and protein levels. This differentiation process is regulated by several cytokines, including BMP2, RUNX2, ALP, OCN and OPN. Among them, BMP2 is one of the most powerful cytokines that promote osteogenic differentiation of mesenchymal cells [56]. BMP2 exhibits this osteogenic action by activating Smad signaling and then regulating the expression and functions of RUNX2 [57–59]. APN treatment increased the expression of BMP2 to three times compare with the control. RUNX2, an important transcription factor in osteogenesis, directly regulates the expression of several osteoblastic genes, including ALP, OCN OPN, and COL1A1 [60,61]. Real-time PCR and Western blot results showed that RUNX2 expression significantly increased after APN stimulation for 7 days compared with the control group. OCN and OPN are the major non-collagenous proteins of bone. They both promote the deposition of calcium phosphate and then regulate bone mineralization. After APN treatment, OCN expression was approximately double that in the control group, and OPN expression was up to six-times that in the control. ALP is an early biochemical marker used to assess osteoblast differentiation and its expression increases in the initial of bone mineralization. ALP catalyzes the hydrolysis of phosphate esters followed by the free transfer of phosphate into the extracellular matrix to promote the calcification of bone. After APN treatment, ALP expression was approximately double that in the control group, suggesting enhanced osteogenic differentiation in hPDLCs. Alizarin Red S showed more calcifying nodules after APN treatment, further demonstrating that APN enhances mineralization in hPDLCs. Consistent with our results, APN has been shown to improve osteogenic differentiation in many other cell lines such as MC3T3-E1, bone marrow stem cells, and human adipose-derived stem cells [16,27,51,62].

We further explored the mechanism underlying APN-induced osteogenesis in hPDLCs. APPL1 is a key regulator of the APN signaling pathway; it binds to APN receptors and mediates downstream signaling molecules. Overexpression of APPL1 increases APN signaling, while inhibition of APPL1 expression decreases it and the APN-mediated downstream signaling [63–66]. Therefore, we examined whether APPL1 is involved in regulating APN-mediated osteogenic differentiation in hPDLCs. We suppressed APPL1 expression with siRNA, which abrogated APN-induced p38 MAPK phosphorylation and osteogenesis-related genes expression. This suggested that APN promotes osteogenic differentiation through the action of APPL1. Then p38 MAPK inhibitor was used to block p38 MAPK pathway, which decreased the expression of osteogenesis-related genes and reduced the formation of mineralized nodules. The results suggested that APN promoted the osteogenesis of hPDLCs. via the p38 MAPK pathway. The similar results was seen in previous study that the inhibition of p38 pathway blocked the APN-induced up-regulation of RUNX2 [67].

In summary, the current study demonstrated that APN suppresses the production of LPS-induced inflammatory factors by suppressing NF-κB phosphorylation and it nuclear translocation in hPDLCs. In addition, APN promotes osteogenic differentiation in hPDLCs via the APPL1 signaling pathway. Therefore, APN appears to be an ideal choice for exerting both anti-inflammatory and osteogenic effects. Our results suggest that APN might serve as a therapeutically beneficial agent for the control of periodontal disease. Topical application of APN at the disease site might suppress the inflammatory response, as well as promote bone formation. However, further investigations are required to demonstrate and validate such therapeutic effects.

## Data Availability

All the data relevant with the present paper are available and we are pleasure to provide original research papers if necessary.

## Abbreviations

APN: adiponectin
APPL1: the adaptor protein containing PH domain, PTB domain, and leucine zipper motif 1
cDNA: complementary DNA
COX: cyclooxygenase
CST: Cell Signaling Technology
FBS: fetal bovine serum
GAPDH: glyceraldehyde-3-phosphate dehydrogenase
HBV: hepatitis B virus
HCV: hepatitis C virus
HIV-1: human immunodeficiency virus-1
hPDLC: human periodontal ligament cell
IL: interleukin
LPS: lipopolysaccharide
MAPK: mitogen-activated protein kinase
NF-κB: nuclear factor κ-B
OCN: osteocalcin
OPN: osteopontin
RIPA: radio immunoprecipitation assay
RUNX2: runt-related transcription factor 2
TNF-α: tumor necrosis factor α
α-MEM: minimum essential medium-α
