# Expression of Concern: Adiponectin inhibits lipoplysaccharide-induced inflammation and promotes osteogenesis in hPDLCs

**DOI:** 10.1042/BSR-20192668_EOC

**Published:** 2021-05-14

**Keywords:** adiponectin, human periodontal ligament cells, inflammation, lipopolysaccharide, osteogenic differentiation

The Editorial Office has been made aware of potential issues surrounding the scientific validity of this paper, hence has issued an expression of concern to notify readers whilst the Editorial Office investigates.

