# Correction: Adiponectin inhibits lipoplysaccharide-induced inflammation and promotes osteogenesis in hPDLCs

**DOI:** 10.1042/BSR-2019-2668_COR

**Published:** 2021-07-02

**Keywords:** adiponectin, human periodontal ligament cells, inflammation, lipopolysaccharide

This Correction follows an Expression of Concern relating to this article previously published by Portland Press.

The Authors of the original article “Adiponectin inhibits lipoplysaccharide-induced inflammation and promotes osteogenesis in hPDLCs” (*Biosci Rep* (2021) 41(3), https://doi.org/10.1042/BSR20192668) would like to correct Figure 5B and 7B, as a duplication of these images had occurred due to an error in their figure making process. The Authors have repeated the experiment and present the correct Figure 7B here. The Authors confirm that this Correction does not change the conclusions of their study.

**Figure 7:**
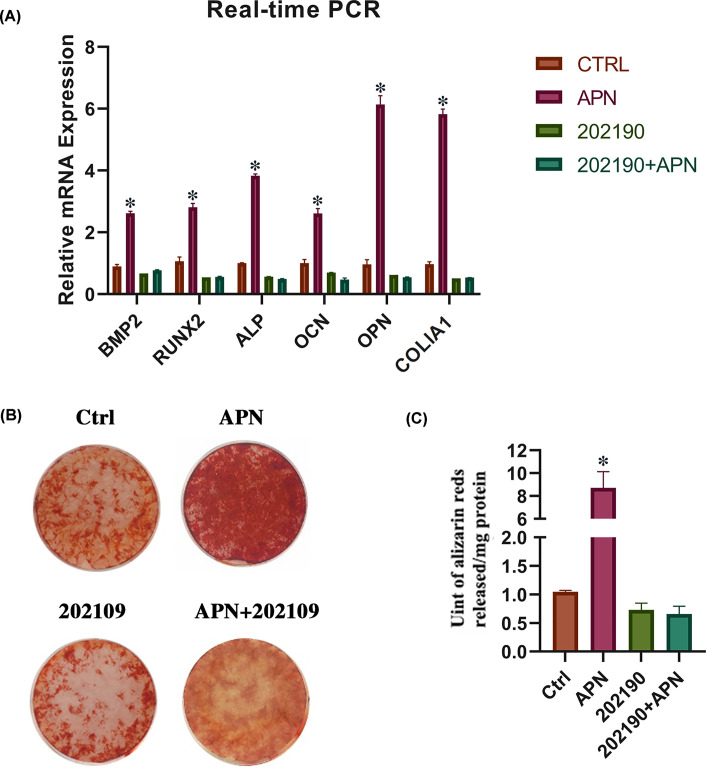
Inhibition of p38 MAPK phosphorylation weaken the osteogenesis induced by APN in hPDLCs (**A**) Relative expression of OPN, COL1A1, OCN, ALP, RUNX2, and BMP2 mRNAs in hPDLCs in different groups (control, APN, 202190, 202190+APN) on day 7 (mean ± SD; *P<0.05 compared with control; experiments performed in triplicate and repeated three times independently). (**B,C**) Representative images of Alizarin Red S staining of hPDLCs in different group (control, APN, 202190, 202190+APN) on day 21 (mean ± SD; *P<0.05 compared with control; experiments performed in triplicate and repeated three times independently).

